# Supplementary material for: Phasor-based hyperspectral snapshot microscopy allows fast imaging of live, three-dimensional tissues for biomedical applications
Source: Commun Biol. 2021 Jun 11;4:721. doi: 10.1038/s42003-021-02266-z (PMC8195998; doi:10.1038/s42003-021-02266-z)
Supplement: Supplementary file 3 — Description of Supplementary Files [file 42003_2021_2266_MOESM3_ESM.pdf]

Description of Additional Supplementary Files

File name: Supplementary Movie M1

Description: 360^0 rotation of the color mapped 3D volume shown in Fig. 3c.

File name: Supplementary Movie M2

Description: 360^0 rotation of the color mapped 3D volume shown in Fig. 5l.

File name: Supplementary Movie M3

Description: 5D rendered time lapse sequence shown in Fig. 5m-q.

File name: Supplementary Data 1

Description: Source Data for Figure 1d.

File name: Supplementary Data 2

Description: Source Data for Figure 1f.

File name: Supplementary Data 3

Description: Source Data for Figure 2c.

File name: Supplementary Data 4

Description: Source Data for Figure 3i.

File name: Supplementary Data 5

Description: Source Data for Figure 5a.

File name: Supplementary Data 6

Description: Source Data for Figure 6h.

File name: Supplementary Data 7

Description: Source Data for Figure 7e.
