# Supplementary figures and images for: Phasor-based hyperspectral snapshot microscopy allows fast imaging of live, three-dimensional tissues for biomedical applications
Source: Commun Biol. 2021 Jun 11;4:721. doi: 10.1038/s42003-021-02266-z (PMC8195998; doi:10.1038/s42003-021-02266-z)

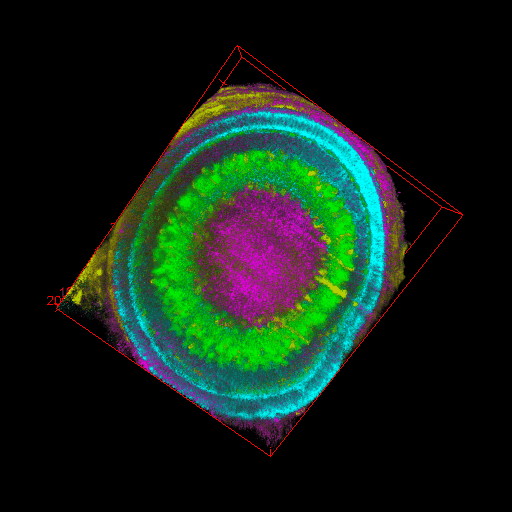

Supplement: Supplementary file 4 — Supplementary Movie M1 [file 42003_2021_2266_MOESM4_ESM.gif]

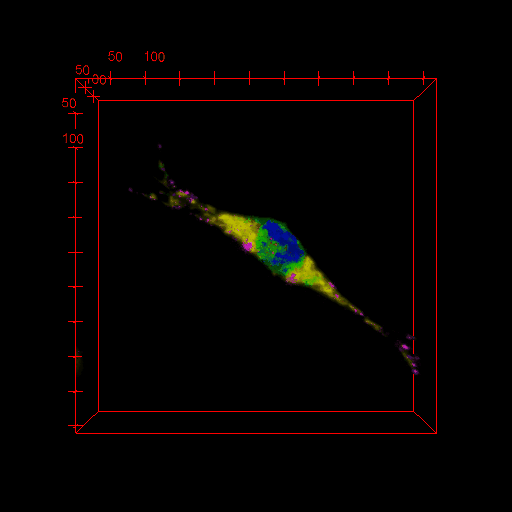

Supplement: Supplementary file 5 — Supplementary Movie M2 [file 42003_2021_2266_MOESM5_ESM.gif]

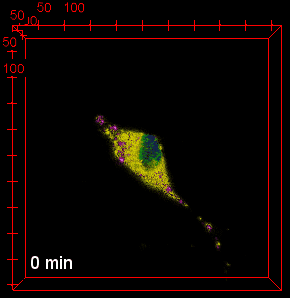

Supplement: Supplementary file 6 — Supplementary Movie M3 [file 42003_2021_2266_MOESM6_ESM.gif]
