# Supplementary material for: Phasor-based hyperspectral snapshot microscopy allows fast imaging of live, three-dimensional tissues for biomedical applications
Source: Commun Biol. 2021 Jun 11;4:721. doi: 10.1038/s42003-021-02266-z (PMC8195998; doi:10.1038/s42003-021-02266-z)
Supplement: Supplementary file 14 — Reporting Summary [file 42003_2021_2266_MOESM14_ESM.pdf]

nature research

Corresponding author(s): Per Niklas Hedde, Enrico Gratton

Last updated by author(s): May 13, 2021

Reporting Summary

Nature Research wishes to improve the reproducibility of the work that we publish. This form provides structure for consistency and transparency in reporting. For further information on Nature Research policies, see our Editorial Policies and the Editorial Policy Checklist.

Statistics

For all statistical analyses, confirm that the following items are present in the figure legend, table legend, main text, or Methods section.

n/a	Confirmed	
☐	☒	The exact sample size (*n*) for each experimental group/condition, given as a discrete number and unit of measurement
☐	☒	A statement on whether measurements were taken from distinct samples or whether the same sample was measured repeatedly
☐	☒	The statistical test(s) used AND whether they are one- or two-sided<br>*Only common tests should be described solely by name; describe more complex techniques in the Methods section.*
☒	☐	A description of all covariates tested
☒	☐	A description of any assumptions or corrections, such as tests of normality and adjustment for multiple comparisons
☐	☒	A full description of the statistical parameters including central tendency (e.g. means) or other basic estimates (e.g. regression coefficient) AND variation (e.g. standard deviation) or associated estimates of uncertainty (e.g. confidence intervals)
☐	☒	For null hypothesis testing, the test statistic (e.g. *F*, *t*, *r*) with confidence intervals, effect sizes, degrees of freedom and *P* value noted<br>*Give P values as exact values whenever suitable.*
☒	☐	For Bayesian analysis, information on the choice of priors and Markov chain Monte Carlo settings
☒	☐	For hierarchical and complex designs, identification of the appropriate level for tests and full reporting of outcomes
☒	☐	Estimates of effect sizes (e.g. Cohen's *d*, Pearson's *r*), indicating how they were calculated

Our web collection on statistics for biologists contains articles on many of the points above.

Software and code

Policy information about availability of computer code

Data collection	MicroManager 1.4, SimFCS 4
Data analysis	SimFCS 4, Origin Lab 2017, MS Excel 2010, Matlab R2019a

For manuscripts utilizing custom algorithms or software that are central to the research but not yet described in published literature, software must be made available to editors and reviewers. We strongly encourage code deposition in a community repository (e.g. GitHub). See the Nature Research guidelines for submitting code & software for further information.

Data

Policy information about availability of data

All manuscripts must include a data availability statement. This statement should provide the following information, where applicable:

- Accession codes, unique identifiers, or web links for publicly available datasets
- A list of figures that have associated raw data
- A description of any restrictions on data availability

Upon request, we will make the data available to other researchers.

Field-specific reporting

Life sciences study design

All studies must disclose on these points even when the disclosure is negative.

Sample size	Fig 2: Solution experiments, 50 images were taken per dye solution.<br>Fig 3,4,7: More than 3 zebrafish were imaged on 3 independent imaging sessions/days.<br>Fig 5: More than 3 different areas were imaged for cell experiments repeated at least 3 times on different days.<br>Fig 6: More than 3 crypts were imaged on 2 independent imaging sessions/days .
Data exclusions	No data was excluded
Replication	In addition to light sheet microscopy, imaging with sine/cosine filters was verified on a home built 2-photon laser scanning microscope (DIVER) and an Olympus X71 customized total internal reflection (TIRF) microscope.
Randomization	NA
Blinding	NA

Reporting for specific materials, systems and methods

We require information from authors about some types of materials, experimental systems and methods used in many studies. Here, indicate whether each material, system or method listed is relevant to your study. If you are not sure if a list item applies to your research, read the appropriate section before selecting a response.

Materials & experimental systems

n/a	Involved in the study	
☒	☐	Antibodies
☐	☒	Eukaryotic cell lines
☒	☐	Palaeontology and archaeology
☐	☒	Animals and other organisms
☒	☐	Human research participants
☒	☐	Clinical data
☒	☐	Dual use research of concern

Methods

n/a	Involved in the study	
☒	☐	ChIP-seq
☒	☐	Flow cytometry
☒	☐	MRI-based neuroimaging

Eukaryotic cell lines

Policy information about cell lines

Cell line source(s)	Mouse Embryonic Fibroblast (MEF)
Authentication	Not authenticated, cell line not relevant for this study
Mycoplasma contamination	Tested negative for mycoplasma
Commonly misidentified lines (See ICLAC register)	NA

Animals and other organisms

Policy information about studies involving animals; ARRIVE guidelines recommended for reporting animal research

Laboratory animals	C57BL/6N mice, obtained from the Knockout Mouse Project (KOMP) repository. Wildtype zebrafish <72 hpf. SoFa zebrafish
Wild animals	NA
Field-collected samples	NA
Ethics oversight	All mouse and zebrafish work was performed in accordance with NIH guidelines and was approved by the Institutional Animal Care and Use Committee (IACUC) of the University of California, Irvine, approval number AUP-17-053.

Note that full information on the approval of the study protocol must also be provided in the manuscript.
